# 1116. Case Study. Bedside Metabolic Cart Monitoring to Assess Caloric Intake for a Burn Patient

**DOI:** 10.1093/jbcr/irag033.290

**Published:** 2026-04-06

**Authors:** Sherwin E Morgan, Naureen Sajwani

**Affiliations:** University of Chicago Burn Center, Chicago, IL; University of Chicago Burn Center, Chicago, IL

## Abstract

**Patient Presentation (age range, injury details, relevant history):**

A 32-year-old male presented to the emergency department following a house fire. He was intubated due to a high suspicion for inhalation injury. He was admitted to the burn unit with partial thickness (second degree flame burns to face and hands, 5% of BSA).

**Clinical Challenges:**

A bronchoscope examination demonstrated, severe inhalation injury; inflammation with friability, copious carbonaceous deposits and bronchorrhea, (GRADE III Inhalation Injury). Blood chemistry results; elevated lactic acid - 26.4 mmol/L and abnormal carboxyhemoglobin - 31%. An abnormal minute ventilation while on mechanical ventilation support made predicting caloric intake (CI) difficult plausibly due to sepsis milieu. The hypermetabolism state made conventional energy intake prediction obfuscated.

**Management Approach:**

After stabilization of the pulmonary status he was change over to a minimal ventilator support breathing mode, Pressure Support (PS), PS - + 5 cm H2O and positive end expiratory pressure (PEEP) - + 8 cm H2O with an FIO2 - 0.40, PaCO2 - 49 mm Hg, pH - 7.35. He was sedated with; (fentanyl and propofol). To assist with CI predictions, bedside indirect calorimetry via metabolic cart (MC) was ordered by the care team, The MC assessment was performed by inserting the airway adapter in the ventilator circuit (VC) between the VC "Y" and artificial airway. The MC measures inspired minute ventilation (tidal volume x breaths/min) plus FIO2 and expired minute ventilation to calculated oxygen consumption (VO2), carbon dioxide production (VCO2), measured resting energy expenditure (mREE) and oxidative substrate utilization by way of respiratory quotient (RQ).

**Outcomes:**

Anthropometrics; weight - 262 lbs, height - 74," BMI - 33.67, BSA - 2.44. average tidal volume - 662 mL, Average Respiratory Rate - 19 breaths/min, minute ventilation (VE) - 12.7 L/min, VCO2 - 491 mL/min, VO2 - 536 mL/min, RQ - 0.92, mREE - 3834 Kcal/day, Predicted REE - 2425 Kcal/day, REE/Pred (%) - 158, CHO/REE (%) - 77, Fat/REE (%) - 23, VD/VT estimated - 0.24. The RQ can identify what substrate is being oxidized; (protein, fat or carbohydrate). The dietary team targeted 2900 Kcal/day using the Harris-Benedict x 1.2 and (calories/kg) formula to calculate CI. A repeat MC one week later demonstrated similar results.

**Lessons Learned:**

Bedside MC requires multidisciplinary team collaboration. Due to unknown stress factors, there may be variations between predicted REE and mREE that may impact outcomes. An REE/Predicted (%) of greater than >110% is an indication of hypermetabolism (158). The mREE and RQ can help the care team make an informed decisions when making recommendations regarding the CI prescription.

**Applicability to Practice:**

This procedure will plausibly assist with improved management of CI surrounding critically ill burn patients with severe inhalation and thermo-injury which may improve overall outcomes. More study is needed to establish the clinical value of MC monitoring in the burn center as the technology has advanced making it more accessible at the bedside.

